# In Situ Observation of the Grain Growth Behavior and Martensitic Transformation of Supercooled Austenite in NM500 Wear-Resistant Steel at Different Quenching Temperatures

**DOI:** 10.3390/ma16103840

**Published:** 2023-05-19

**Authors:** Zhongbo Li, Qing Yuan, Shaopu Xu, Yang Zhou, Sheng Liu, Guang Xu

**Affiliations:** 1 State Key Laboratory of Refractories and Metallurgy, Key Laboratory for Ferrous Metallurgy and Resources Utilization of Ministry of Education, Wuhan University of Science and Technology, Wuhan 430081, China; lzb7460377@163.com (Z.L.); liusheng@wust.edu.cn (S.L.); xuguang@wust.edu.cn (G.X.); 2 Nanyang Hanye Special Steel Co., Ltd., Nanyang 474500, China; lcb-9999@163.com (S.X.); zy18203778277@126.com (Y.Z.)

**Keywords:** in situ observation, austenite, martensite, twins, quenching temperature

## Abstract

In situ observations of the austenite grain growth and martensite transformations in developed NM500 wear-resistant steel were conducted via confocal laser scanning high-temperature microscopy. The results indicated that the size of the austenite grains increased with the quenching temperature (37.41 μm at 860 °C → 119.46 μm at 1160 °C) and austenite grains coarsened at ~3 min at a higher quenching temperature of 1160 °C. Furthermore, a large amount of finely dispersed (Fe, Cr, Mn)_3_C particles redissolved and broke apart at 1160 °C, resulting in many large and visible carbonitrides. The transformation kinetics of martensite were accelerated at a higher quenching temperature (13 s at 860 °C → 2.25 s at 1160 °C). In addition, selective prenucleation dominated, which divided untransformed austenite into several regions and resulted in larger-sized fresh martensite. Martensite can not only nucleate at the parent austenite grain boundaries, but also nucleate in the preformed lath martensite and twins. Moreover, the martensitic laths presented as parallel laths (0~2°) based on the preformed laths or were distributed in triangles, parallelograms, or hexagons with angles of 60° or 120°.

## 1. Introduction

Wear, fracture and corrosion are usually the main failure modes during the service of metal materials. Wear will not directly cause the failure of metal parts, but equipment parts are difficult to repair due to wear. Besides, frequent replacement significantly reduces the working efficiency and service life of equipment, thus leading to a large amount of material and energy loss [1,2,3,4]. At present, among the metal wear-resistant materials, austenitic high manganese (Mn) steel, high chromium (Cr) cast iron and low alloy wear-resistant steel are the most widely used. Among them, austenitic high manganese steel has a surface austenitic structure, quickly producing work hardening by a phase transition under strong extrusion or impact. The core of austenitic high manganese steel still retains good toughness and plasticity because of the austenitic structure [5,6,7]. However, the wear resistance of austenitic high manganese steel is relatively low under low or medium stress conditions, which severely limits their application scope in the wear-resistant materials field [8]. High chromium cast iron, as a second-generation wear-resistant material, is currently recognized as the best wear-resistant material [9,10,11]. A lot of non-network carbide M_7_C_3_ with a hardness of 1600 HV is precipitated in high chromium cast iron and the toughness and plasticity is better than that of white cast iron [12]. Therefore, mechanical equipment made of high chromium cast iron can meet the needs of long-term wear resistance in complex environments. However, due to the large number of valuable elements such as Cr and nickel (Ni), the production of high chromium cast iron is complex, which also increases the production cost and limits its wide application in industrial production [13]. In view of the many problems regarding the service and production of the above two steels, low-alloy wear-resistant steel has gradually become the research topic of the new generation of wear-resistant metal materials [14,15,16,17,18].

Various wear-resistant steel products are made by Chinese iron and steel manufacturers, among which the production technology of the products below NM400 is relatively mature. Tempered martensite is obtained by off-line re-austenitizing after rolling to improve the strength hardness, and then the toughness is improved by subsequent tempering. Martensite has ultra-high strength and hardness among the different microstructures in steel, and it is usually selected as an important microstructure in the production of ultra-high strength steel. Therefore, among all the kinds of low-alloy wear-resistant steels, martensitic wear-resistant steel is promising. The wear resistance of martensite mainly relies on its high hardness, but the wear resistance under high impact is erratic due to its poor toughness. Therefore, many studies on martensitic wear-resistant steel have focused on improving its toughness and plasticity [19,20,21]. It has been revealed that martensitic laths and blocks are the organizational units affecting strength and hardness, while martensitic packets affect the plasticity and toughness [22,23]. In addition, Liang et al. found that crack propagation could be effectively inhibited by using smaller sizes and angles of the martensitic packet [24]. The main structure control unit affecting fracture, the size of martensitic block, was identified by Inoue et al. through a study on the cleavage fracture of tempered martensitic steel [25]. Moreover, some scholars improved the fracture toughness and elongation of martensitic steel by optimizing the composition and heat treatment process, so that about 30% residual austenite was obtained at room temperature [26].

In addition, the wear resistance of martensitic steel had been studied extensively. Liang et al. reported that low-alloy martensitic wear-resistant steel exhibited better wear resistance under moderate impact wear, and its comprehensive mechanical property was more than twice that of austenitic high manganese steel [27]. Cao et al. prepared Ti-Cr-B (boron) microalloyed high-strength wear-resistant steel with tempered martensite, in which a high dislocation density and tempered carbide precipitation hardened the matrix [28]. In the work of Ma et al., they found that the solid solution carbon content in the martensitic structure was a direct factor affecting the wear resistance and subsurface hardness [29].

Research on high-grade low-alloy wear-resistant steel is insufficient. A high-grade NM500 wear-resistant steel is presented in the present study. Martensite transformation greatly influenced the microstructure and properties of the low-alloy wear-resistant steel, and the quenching temperature and the subsequent cooling also played a decisive role in these properties. However, few studies about NM500 wear-resistant steel have studied the relationship between grain growth and martensite transformation. Moreover, a dynamic investigation into austenite grain growth and martensite transformation in NM500 wear-resistant steel has not been conducted. Therefore, the phase transformation behavior of NM500 wear-resistant steel in a continuous cooling process was analyzed by confocal laser scanning high-temperature microscopy (CSLM). The novelty of the present work is summarized in two aspects: (1) the grain growth behavior of high grade NM500 wear-resistant steel at two quenching temperatures was first recorded and compared and (2) the martensite transformations in different austenite grains were dynamically analyzed. The results of the present study will provide a reference to understand austenite grain growth and martensite transformation at different quenching temperatures.

## 2. Experimental Procedures

**Figure 1:**
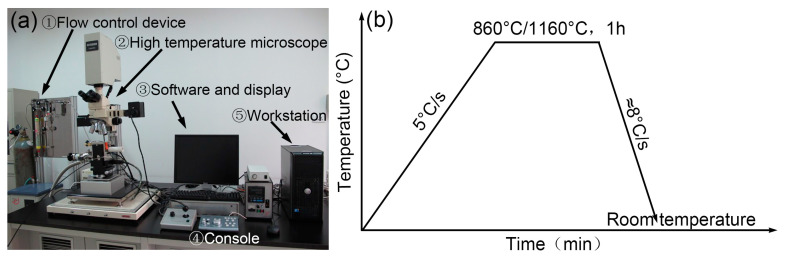
(**a**) The confocal laser scanning high-temperature microscope and (**b**) in situ observation process.

Figure 1 shows the VL2000DX-SVF17SP confocal laser scanning high-temperature microscope and the corresponding quenching process. This CSLM equipment consists of a flow control device, a console, a display, high temperature microscope, etc., which can observe and capture all kinds of physical and metallurgical phenomena in real time. It studies the dynamic process at a high resolution in real time through high-speed laser scanning imaging. A higher automation degree was achieved through digital image information storage and processing technology. The light source of CSLM was a blue laser, whose wavelength and resolution were about 410 nm and 0.25 μm, respectively. Various phases emerged under the effect of thermal etching, rather than chemical corrosion.

**Figure 2:**
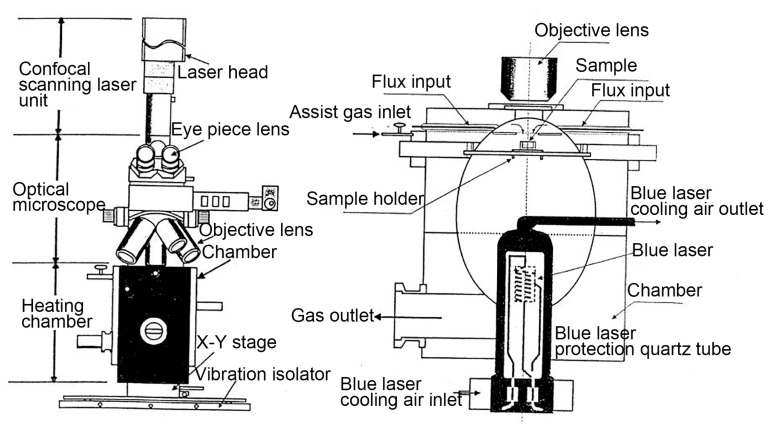
The structure of a confocal laser scanning high-temperature microscope.

The experimental steel was a developed high-grade NM500 steel with the chemical composition Fe-0.23C-0.20Si-1.49Mn-1.15Cr-0.25Ni-0.37(Nb+V+Ti+Mo)-0.022Cu-0.00174B-0.01P-0.002S (wt.%). Figure 2 illustrates the structure of the confocal laser scanning high-temperature microscope. A small cylindrical sample with dimensions of Φ 6 mm × 5 mm was finish machined, and the two faces of the sample were polished until a mirror surface was obtained. Subsequently, the sample was placed into the Al_2_O_3_ crucible for in situ observation. Before the experiment, the sample chamber was vacuumed to 6 × 10^−3^ Pa, and then argon gas was introduced to prevent sample oxidation. Microstructure evolution was recorded throughout the whole process with a recording frequency of 5 photos/s. Figure 1b demonstrates the heating process with two different quenching temperatures. Firstly, the samples were reheated to 860 °C and 1160 °C, respectively, at a rate of 5 °C/s and then held for 1 h. Subsequently, the maximum cooling rate was applied to cool the specimens to room temperature after thermal holding to simulate the quenching process. The heating rate of 5 °C/s was determined by an empirical value considering its small size. Two different quenching temperatures of 860 °C and 1160 °C were chosen according to the minimum and maximum tempering parameters in industrial production. The holding time of 1h was utilized also based on the requirements of industrial production. It should be noted that the average cooling rate during quenching was estimated to be only 8 °C/s due to the low cooling ability at a low temperature range. However, the cooling rate before martensite transformation could reach 15 °C/s, totally ensuring the martensite transformation. In addition, to facilitate the analysis of grain size evolution, the eyepiece was manually changed during the in situ observation experiment. An appropriate magnification was selected due to the different size of parent austenite grains (PAGs) at 860 °C and 1160 °C. Meanwhile, the precipitates quenched at 1160 °C were identified by transmission electron microscopy (TEM) using a thin film specimen.

## 3. Results and Discussion

### 3.1. Austenite Nucleation

**Figure 3:**
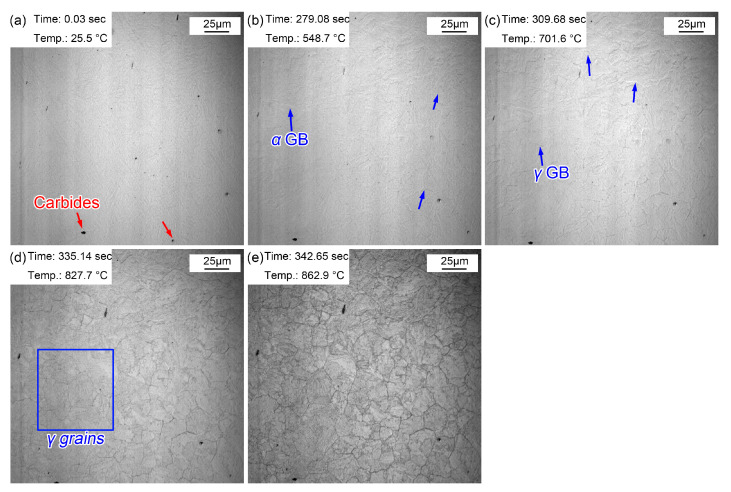
Morphology variations from room temperature to 860 °C. (**a**) 25.5 °C, before heating; (**b**) 548.7 °C, initial grain boundaries appeared; (**c**) 701.6 °C, austenization began; (**d**) 827.7 °C, obvious austenite grains; (**e**) 862.9 °C, the preset quenching temperature.

Figure 3 shows the morphologic changes from room temperature to the preset temperature of 860 °C. Some particles were present on the surface of the sample, and these dark particles were (Fe, Cr, Mn)_3_C precipitates (Figure 3a) determined by the following results. Due to the increased amount of Cr and Mn, (Fe, Cr, Mn)_3_C precipitates dominated in the as-received steel treated by hot rolling and subsequent air cooling. When the temperature was increased to 548.7 °C, some corrugated folds appeared on the sample surface. These folds correspond to the grain boundaries of the initial ferrite (pearlite), which gradually emerged under the effect of thermal etching (Figure 3b). As the temperature increased to 701.6 °C, another corrugated fold gradually covered the grain boundaries of the existing ferrite (Figure 3c). Additionally, this corrugated fold became more and more clear and gradually formed the grain boundaries of polygonal grains as the temperature rose to 827.7 °C (Figure 3d) and 862.9 °C (Figure 3e). It was inferred that the corrugated fold at 701.6 °C was an austenitic grain boundary, that is, the A_c1_ temperature (the beginning temperature at which the pearlite transforms to austenite during the heating process) was about 701.6 °C when the steel was reheated at 5 °C/s. The measured A_c1_ temperature of this experimental steel was about 658 °C using a thermal simulated test, a little lower than that obtained via in situ observations. The measured A_c1_ temperature was obtained with a very slow heating rate (about 0.1 °C/s), and the A_c1_ temperature increased with the increase in the heating rate (5 °C/s). Moreover, austenization process of the sample finished more quickly at a higher heating rate. The austenization process completed at 862.9 °C (Figure 3e), but the grain boundary morphology of the initial microstructure remained. Meanwhile, the visible (Fe, Cr, Mn)_3_C precipitates became clearer and their size increased.

Austenite transformation is related to the nucleation rate and the growth rate, and can be expressed as Equations (1) and (2) [30,31]:

(1) $$N=f_{N}exp\;(-Q_{N}/K∆T)$$

(2) $$G=f_{G}exp\;(-Q_{G}/K∆T)$$

where *N* is the nucleation rate, *G* is the growth rate, $Q_{N}$ and $Q_{G}$ are the nucleation and growth activation energies, respectively, $f_{N}$ and $f_{G}$ are the impact factors between structure and nucleation with growth, respectively, and ∆T is the superheat. This equation reveals that the superheat increases with the increase in the heating rate, which increases the nucleation and growth rates of the austenitic transformation. Therefore, the rate of austenitic transformation increased significantly, the time required from initial austenization to complete austenization was greatly reduced, and thus the required phase transition interval was correspondingly reduced. In addition, the transformation of steel during continuous heating is equivalent to the accumulation of countless isothermal transformations. The relationship between the isothermal incubation period and the transition temperature can be established using Scheil’s superposition principle [32]:

(3) $$\sum_{i=1}^{i=n}\frac{∆t}{A_{i}}=1$$

Differential Equation (4) is obtained when ∆*t* is small enough.

(4) $$\int_{t=0}^{t=t_{n}}\frac{dt}{A(T)}=1$$

where ∆*t* and *dt* are the transformation time at temperature *T* and *A_i_* and *A*(*T*) are the corresponding incubation periods. The relationship between the incubation period and the transition temperature is shown in Equation (5):

(5) $$\int_{T_{1}}^{T_{s}}\frac{dt}{A\left(T\right)}=\int_{T_{1}}^{T_{s}}\frac{1}{A\left(T\right)}\cdot \frac{1}{\frac{dT}{dt}}\cdot dT=\int_{T_{1}}^{T_{s}}\frac{1}{A\left(T\right)}\cdot \frac{1}{v}\cdot dT=1$$

The relationships between the transformation rate, *C*, transformation beginning and ending temperatures, *T_s_* and *T_f_*, and the heating rate, *v*, are interpreted by Equations (6) and (7), where the transformation volume is *f*.

(6) $$c=\frac{df}{dt},v=\frac{dT}{dt}$$

(7) $$\int_{t=0}^{t=t_{n}}\frac{df}{dt}dt=\int_{T_{s}}^{T_{f}}\frac{df}{dt}\cdot \frac{dT}{v}=\int_{T_{s}}^{T_{f}}\frac{C}{v}\cdot dT=1$$

where *T*_1_ is the equilibrium temperature and *t_n_* is the time to the transformation ending temperatures *T_f_*. This equation proves that with the increase in the heating rate, both the initial temperature and the end temperature of the phase transition increase. In addition, the dissolution and diffusion of carbonitrides is inevitable during the austenization process of experimental steel, and atoms migrate between phases through the diffusion mechanism. With the increase in the heating rate, the diffusion of carbon and alloying elements at the equilibrium temperature decreases, thus increasing the austenitic transition temperature. In the process of continuous heating, with the increase in temperature, the diffusion coefficient and diffusion rate of atoms increase greatly, so the driving force of the austenite phase transformation is enhanced.

**Figure 4:**
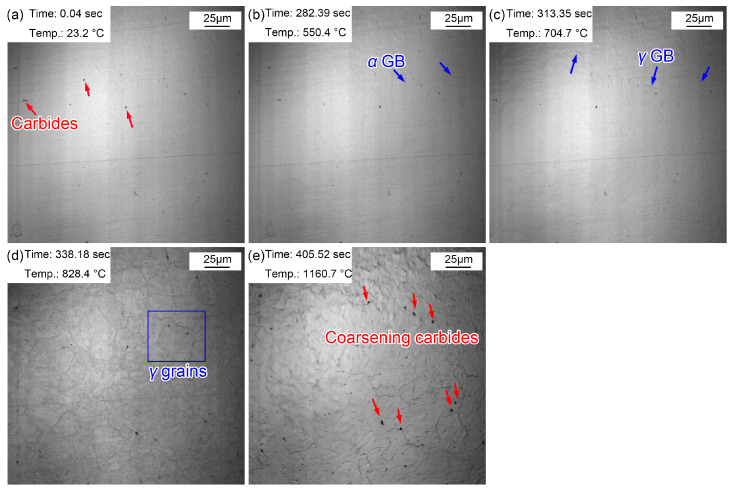
Morphology variations from room temperature to 1160 °C. (**a**) 23.2 °C, before heating; (**b**) 550.4 °C, initial grain boundaries appeared; (**c**) 704.7 °C, austenization began; (**d**) 828.4 °C, obvious austenite grains; (**e**) 1160.7 °C, the preset quenching temperature.

**Figure 5:**
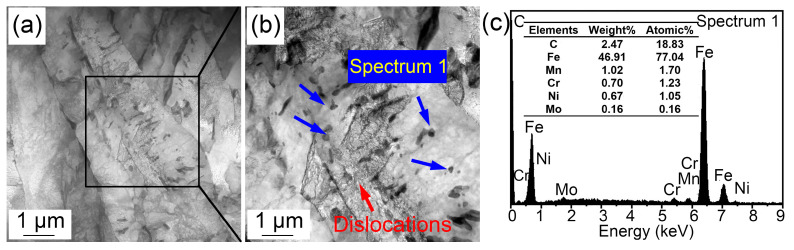
(**a**,**b**) TEM images and (**c**) energy spectrum showing (Fe, Cr, Mn)_3_C particles at 1160 °C.

The morphological variations from room temperature to 1160 °C are displayed in Figure 4. Some (Fe, Cr, Mn)_3_C particles appeared on the sample surface (Figure 4a) and corrugated folds appeared at 550.4 °C (Figure 4b). Another corrugated fold gradually covered the grain boundaries of the existing ferrite structure at 704.7 °C (Figure 4c). The corrugated fold became more and more clear and gradually formed the grain boundaries of polygonal grains at 828.4 °C (Figure 4d). The A_c1_ temperature was basically the same as that in specimen reheated to 860 °C. This is because the heating processes of the two samples were the same before heating to 860 °C. However, when the sample was reheated to 1160 °C (Figure 4e), more large-sized grains appeared and the grain boundaries became sharper and clearer. In addition, the number of clearly visible (Fe, Cr, Mn)_3_C particles increased significantly, and they gradually became more coarse. This can be explained by the gradual dissolution of some invisible finely dispersed (Fe, Cr, Mn)_3_C particles at 1160 °C. The austenite grain boundary mobility increased, quickly resulting in the coarsening of austenite grains. The increased amount of visible (Fe, Cr, Mn)_3_C particles was attributed to the ripening of more micro/nano (Fe, Cr, Mn)_3_C particles at high temperatures. Compared with the sample quenched at 860 °C, the austenite grains quenched at 1160 °C clearly coarsened. Figure 5 shows the (Fe, Cr, Mn)_3_C particles in the specimen quenched at 1160 °C by TEM and the related energy spectrum. (Fe, Cr, Mn)_3_C particles was a compound of cementite (Fe_3_C) with other alloy elements. A few microalloy elements such as Ti, V, and Mo were captured due to their increased solvation at higher temperatures. In addition, apparent quenching dislocations were observed in the lath martensite.

### 3.2. Austenite Growing

**Figure 6:**
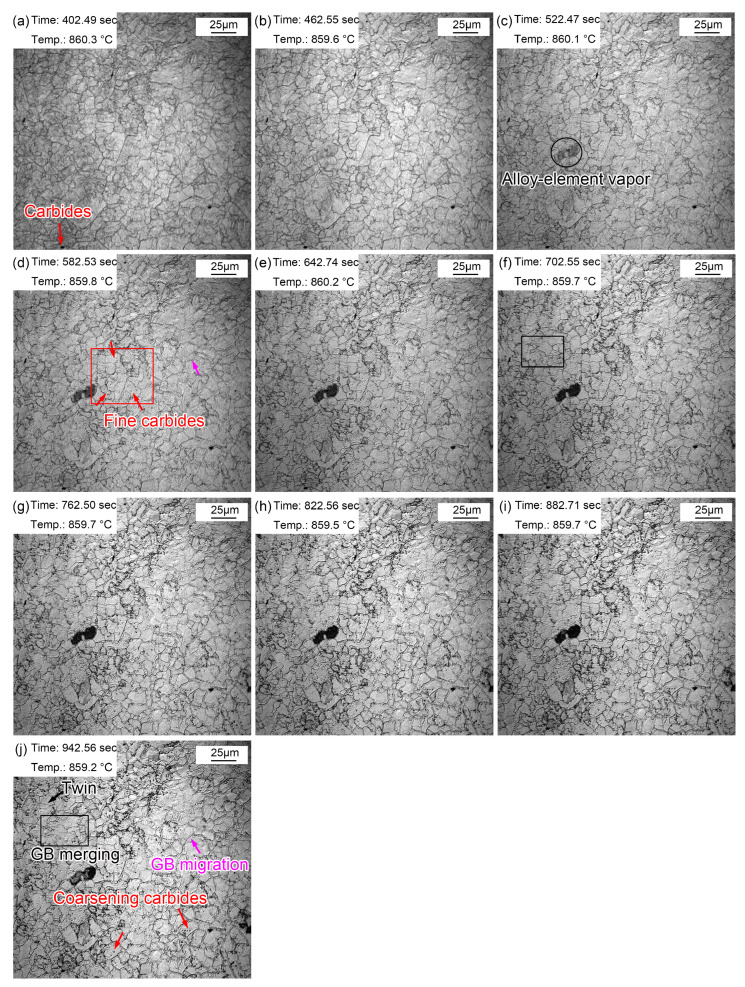
Morphologies during thermal holding at 860 °C from 1~10 min. (**a**) 1 min; (**b**) 2 min; (**c**) 3 min; (**d**) 4 min; (**e**) 5 min; (**f**) 6 min; (**g**) 7 min; (**h**) 8 min; (**i**) 9 min; (**j**) 10 min.

Figure 6 shows the morphologic changes from 1~10 min with a time interval of 1 min when the quenching temperature was 860 °C. Compared with the morphology after just reaching the preset temperature, the grain boundaries of austenite grains were clearer after 1 min (Figure 6a). This is because the grain boundary grooves are more easily exposed after longer thermal etching. In addition, the austenite grain boundaries were narrow and straight with a grain boundary angle of 120°. Some local small grains gradually merged into large ones, as shown in the rectangle in Figure 6f,j. In addition, the austenite grain boundaries expanded and migrated to form large grains, as shown by the pink arrows in Figure 6d,j. The gradual merging of small grains and the migration of some grain boundaries indicated a unconspicuous growth process and trend.

During the thermal holding process, a fog-like substance shown by the oval in Figure 6c appeared. The fog-like substance gradually turned black, and then subsequently disappeared. This dark mist is the vapor of alloying elements, which tends to steam outward from the steel matrix when reheated. A similar phenomenon was also reported in the research of Lan et al. [33]. Manganese (Mn) volatilization was determined by a simultaneous thermal analysis, and they clarified that Mn tended to migrate to the substrate surface and volatilize when the temperature was high enough. In addition to the clearly visible precipitates at the beginning of reheating, many fine (Fe, Cr, Mn)_3_C particles also appeared in the austenite grains during thermal holding, as shown in Figure 6d. These fine (Fe, Cr, Mn)_3_C particles gradually appeared as some of the unprecipitated (Fe, Cr, Mn)_3_C particles matured and emerged. The (Fe, Cr, Mn)_3_C particles matured during thermal holding, as shown in Figure 6j.

**Figure 7:**
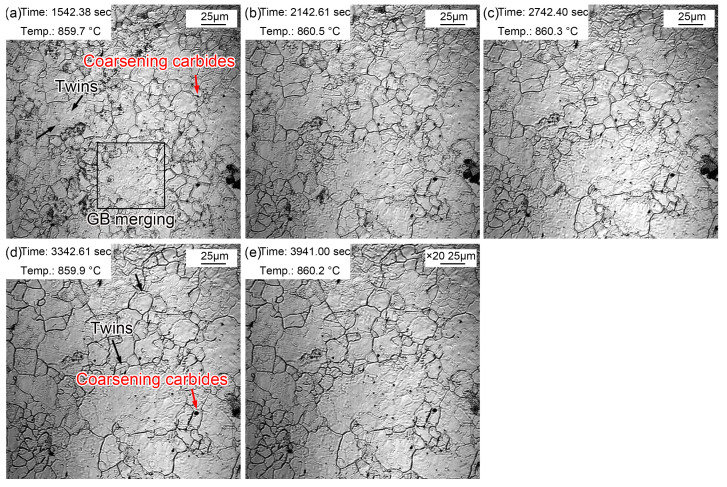
Morphologies during thermal holding at 860 °C from 20~60 min. (**a**) 20 min; (**b**) 30 min; (**c**) 40 min; (**d**) 50 min; (**e**) 60 min.

The morphologic variation in a time interval of 10 min from 20~60 min at the quenching temperature of 860 °C is exhibited in Figure 7. The coarsening of (Fe, Cr, Mn)_3_C particles was more obvious, and there were more areas where small grains merged into large grains. In addition, twins could be observed in austenite grains (Figure 7a). The existing fog-like steam gradually volatilized and disappeared during thermal holding, while it appeared in other areas. This may be explained by the uneven distribution of some alloying elements such as Mn. In addition, austenite grain coarsening was obvious during thermal holding, in which the proportion of small grains decreased gradually. Moreover, and the intramural twins were more clearly visible (Figure 7e). The intracrystalline twins can be considered as annealing twins [34]. There were more alloying elements in the experimental steel, which significantly reduced the stacking fault energy. Compared with ordinary carbon steel, intracrystalline twins are more likely to occur in alloyed steels. The appearance of twins segregated and refined the grains, thus increasing the resistance of dislocation movement and strengthening the steel.

**Figure 8:**
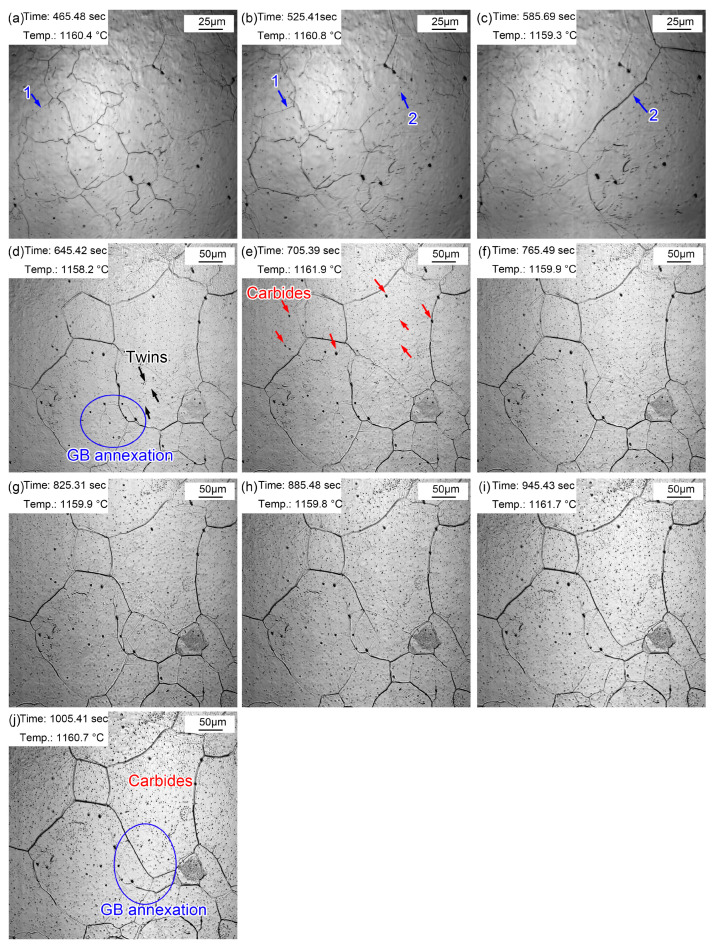
Morphologies during thermal holding at 1160 °C from 1~10 min. (**a**) 1 min; (**b**) 2 min; (**c**) 3 min; (**d**) 4 min; (**e**) 5 min; (**f**) 6 min; (**g**) 7 min; (**h**) 8 min; (**i**) 9 min; (**j**) 10 min.

Figure 8 presents the morphologic evolution from 1~10 min at the quenching temperature of 1160 °C. The austenite grains were much clearer after holding at 1160 °C for 1 min (Figure 8a) as a result of continuous thermal etching. The migration of grain boundaries was obvious during holding, as shown in Figure 8a,b (blue arrow 1) and Figure 8b,c (blue arrow 2). Part of the original grain boundaries gradually faded away during grain boundary migration, and the old ones were gradually filled in. In addition, except for the outward expansion of grain boundaries, small grains were partitioned by surrounding large grains, as shown in the oval in Figure 8d. Alloy element steam, as shown in Figure 7c, also appeared in the specimen reheated to 1160 °C. In addition, many annealing twins traversing or occupying the whole grain were captured in the austenite grains. (Fe, Cr, Mn)_3_C particles ripened during the holding process, as shown in Figure 8j. The gradual appearance of fine (Fe, Cr, Mn)_3_C particles was attributed to (Fe, Cr, Mn)_3_C ripening at 1160 °C, which was captured by limited magnification. Compared with the grain morphology at 860 °C for 20~60 min, the grain size was obviously coarsened at 1160 °C, and there were many dense fine (Fe, Cr, Mn)_3_C particles inside the grains. 

The coarsening of austenite grains at 1160 °C is related to the redissolution of (Fe, Cr, Mn)_3_C particles. Firstly, the atomic size of Cr/Mn/Ti is very different to Fe, which causes a certain solute atomic dragging effect. Reconcentration of a large number of solute atoms such as Cr, Mn, vanadium (V), and titanium (Ti) at the grain boundaries or subgrain boundaries could prevent the migration of grain boundaries and thus inhibit recrystallization. In addition, (Fe, Cr, Mn)_3_C particles were preferentially precipitated at the grain boundaries and dislocation lines, pinning the austenite grain boundaries and hindering the growth of austenite grains. Grain boundary migration caused austenite grain growth. The surface energy increased when the grain boundaries contacted the (Fe, Cr, Mn)_3_C particles. Only when the thermal activation energy was greater than the increased surface energy were the (Fe, Cr, Mn)_3_C particles cut or bypassed by the grain boundary. Therefore, the (Fe, Cr, Mn)_3_C particles significantly slowed down the formation of austenite and prevented the growth of grains. Similar observations were made in the work of G. Khalaj et al., where they established a model to predict the austenite grain size in Nb/Ti microalloyed steel [35]. Since the solute concentration around small particles was greater than those around large particles, the solute atoms spread from small particles to large particles, resulting in the redissolution of small particles and the growth of larger particles. Therefore, the fine precipitates gradually redissolved and continuously formed large size carbonitride particles when the holding time was long enough at 1160 °C.

**Figure 9:**
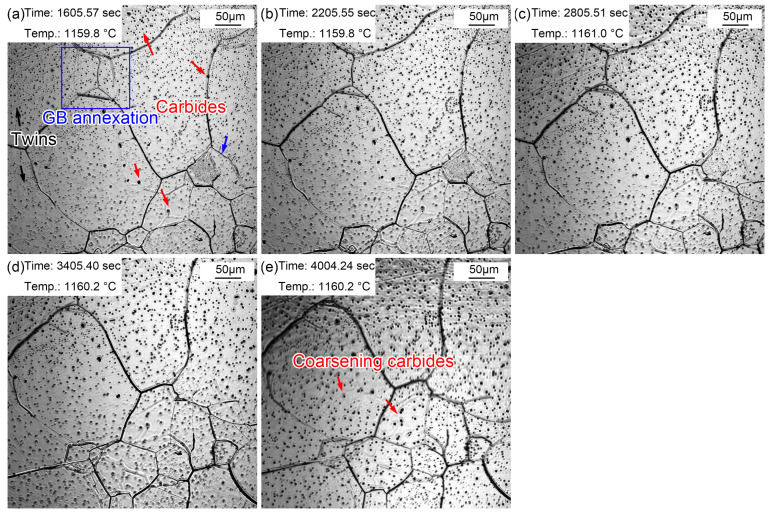
Morphologies during thermal holding at 1160 °C from 20~60 min. (**a**) 20 min; (**b**) 30 min; (**c**) 40 min; (**d**) 50 min; (**e**) 60 min.

Figure 9 displays the morphologic changes with a time interval of 10 min from 20 to 60 min at 1160 °C. Dense, small (Fe, Cr, Mn)_3_C particles formed in the austenite grains. This signifies that the coarsening of (Fe, Cr, Mn)_3_C particles was more obvious compared to that during the holding time of 10 min. In addition, the grain boundaries of small-size austenite were gradually absorbed by the surrounding large-size austenite. In addition, apparent twins were observed in austenite grains (Figure 9a). Furthermore, austenite grain coarsening still occurred during holding from 20 to 60 min, in which the proportion of small grains further decreased. The intra twins were more clearly visible (Figure 9e) because of the larger austenite grains.

### 3.3. Grain Size

**Figure 10:**
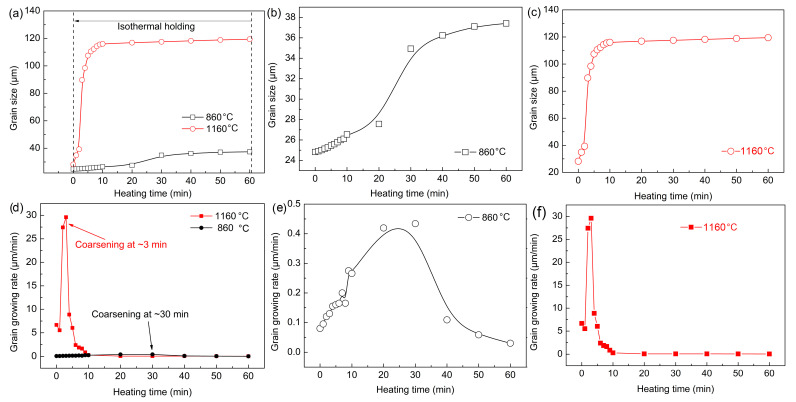
(**a**–**c**) Grain size and (**d**–**f**) growth rate of austenite from formation to thermal holding at different quenching temperatures.

Figure 10 summarizes the grain size and growth rate of austenite at different quenching temperatures. Enough grains were present to ensure an improvement in the accuracy of the statistical process. Grains less than half the average size grain were not counted, and grains larger than half the average size grain were considered. There was little difference in the austenite grain size during the reheating stage before the preset quenching temperatures. However, the austenite grain size at 860 °C was always smaller than that at 1160 °C. Figure 10b shows the growth trend of austenite grains with time at 860 °C. The growth process was relatively slow, and the obvious coarsening of austenite grains was complete after holding for about 30 min. The growth process of austenite grains was very rapid and intense, and the coarsening of austenite grains was completed within 10 min at 1160 °C. Austenite grain growth rate curves at different quenching temperatures were obtained through the first derivative (Figure 10d–f). The austenite grain growth rate was significantly faster at 1160 °C. In addition, when the quenching temperature was 1160 °C, the maximum austenitic growth rate appeared at the holding time of ~3 min, whereas it occurred at ~30 min at 860 °C. 

It can be concluded that the austenite grains coarsened in a short time and the coarsening rate was higher at 1160 °C. This is because the austenite grain boundary migration ability was increased at a higher temperature. The atomic diffusion process was more rapid, and part of the grain boundary faded and disappeared more easily. In addition, many small dispersed (Fe, Cr, Mn)_3_C particles redissolved and ripped, which led to a significant decrease in the migration ability of austenite grain boundaries. Furthermore, the growth rate began to decrease gradually when austenite grains coarsened extensively. This was because the energy for grain growth can no longer be provided as the heating temperature was unchanged, and the redissolution and breaking of the (Fe, Cr, Mn)_3_C particles was basically resolved. Consequently, the pinning effect of (Fe, Cr, Mn)_3_C particles on austenite grain boundaries was stabilized, so the austenite coarsening gradually weakened.

### 3.4. Martensite Transformation

**Figure 11:**
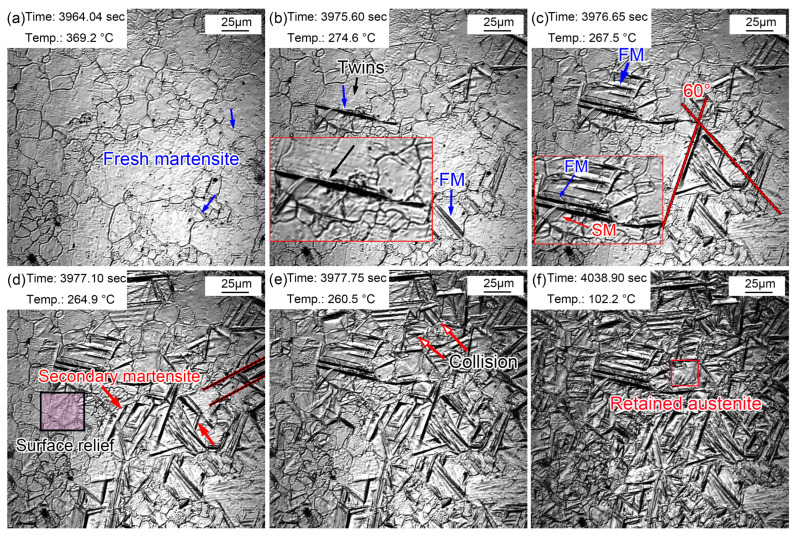
Martensite transformations of supercooled austenite quenched at 860 °C. (**a**) 369.2 °C, martensite appeared; (**b**) 274.6 °C, martensite increased; (**c**) 267.5 °C, martensite nucleated and grew at the twins; (**d**) 264.9 °C, SM and surface relief; (**e**) 260.5 °C, martensitic lath collisions; (**f**) 102.2 °C, martensitic transformation stopped and residual austenite formed.

The above-mentioned austenite grain growth rules indicated that the austenite grain size greatly varied at different quenching temperatures. It has been pointed out that the austenite grain size affected the martensitic transformation temperature and the phase transformation behavior of supercooled austenite during cooling. Figure 11 shows the martensitic transformation during cooling in the sample quenched at 860 °C. Lath martensite appeared, as shown by the blue arrow in Figure 11a, when the temperature decreased to 369.2 °C. This martensite was primary martensite, also called fresh martensite (FM). The martensitic phase transition point (M_s_) of the sample was about 369.2 °C, while the M_s_ temperature of this steel was determined to be 340 °C via a thermal simulation experiment. The effective M_s_ was obtained via a thermal simulation experiment through the overall volume expansion effect of the martensitic transformation, while in situ observation determined the M_s_ just according to the temperature at which the martensite appeared in one certain grain. Generally speaking, the M_s_ determined by in situ observations is higher than that reflected in thermal simulation experiments. This is because the martensitic transformation does not start at the same time in all grains, although the nucleation and growth of martensite explosively proceeded following this. In addition, martensite nucleated from the grain boundary and grew in between grains until stopping at the grain boundary. More and more lath martensite explosively appeared as the temperature decreased, and most lath martensite traversed the entire grain. Furthermore, some lath martensite was found to nucleate and grow from the twins (Figure 11b). Since the formed martensite stimulated the nucleation of the surrounding untransformed austenite, the austenite nucleated and grew in parallel after this trigger. Therefore, the lath martensite grew in a parallel manner in some austenite grains. The lath martensite appeared simultaneously with an angle of 60° at 267.5 °C. In addition, some lath martensite simultaneously formed parallel to each other. More FM was observed as the temperature continued decreasing accompanied by secondary martensite (SM). SM refers to martensite with slightly thin laths formed around FM, which appeared at a certain angle with FM (Figure 11c). More and more surface reliefs due to martensitic transformations gradually appeared at the PAG boundaries (Figure 11d). Most martensite stopped growing when they encountered grain boundaries, and some martensite met each other, which also stopped the growth of lath martensite (Figure 11e). The nucleation and growth of martensite were very weak when the temperature approached room temperature. Most martensite transformations finished within 13 s, and the rate of martensitic transformations gradually slowed down. However, the distortion caused by martensitic transformations prevented martensitic transformations in the surrounding austenite. Small parts of the regions were retained as residual austenite, in which the sharing of elements such as carbon in ferrite to residual austenite was mainly completed (Figure 11f). The growth rate of lath martensite was relatively fast, but the growth rate of longitudinal lath martensite was faster than that of lateral lath martensite. Although the martensitic transformations explosively proceeded, the martensitic transformations were not simultaneous. Martensitic transformations selectively started in PAGs, but this selective process was very short. Nevertheless, the temperature of the sample may remain unchanged or even slightly increase during the cooling process since martensitic transformations release more latent heat of transformation. Therefore, isothermal martensite formation was inevitable. This latent heat caused by martensitic transformations was also one of the reasons for the selective initiation of martensitic transformations. The supercooling degree became smaller at a constant or slightly increased temperature; thus, martensitic transformations were inhibited. In addition, the selective initiation of martensitic transformations was also related to the distortion caused by martensitic transformations. Martensitic transformations in untransformed austenite were strongly inhibited by the surrounding martensitic transformations.

**Figure 12:**
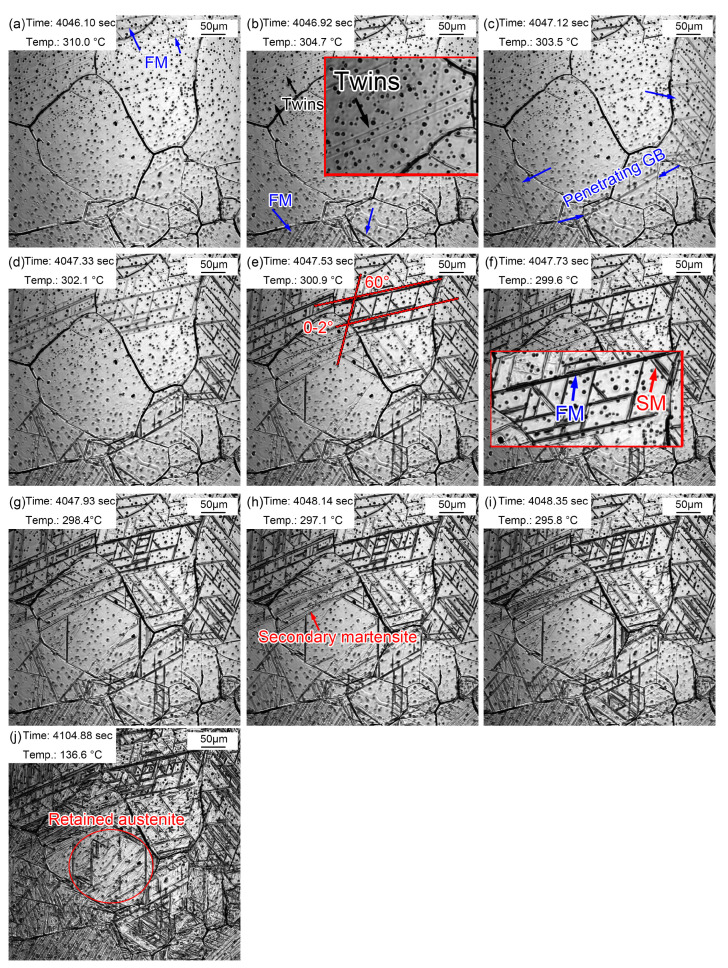
Martensite transformation of supercooled austenite quenched at 1160 °C. (**a**) 310.0 °C, martensite appeared; (**b**) 304.7 °C, martensite increased; (**c**) 303.5 °C, martensite traversed grain boundaries; (**d**) 302.1 °C, martensite increased and appeared at 60° angles; (**e**) 300.9 °C, martensitic packet; (**f**) 299.6 °C, SM increased; (**g**–**i**) 298.4 °C, explosive martensite; (**j**) retained austenite.

The martensitic transformation of the sample quenched at 1160 °C is displayed in Figure 12. The martensitic phase transition point, M_s_, was about 310.0 °C (Figure 11a), which was lower than that in the sample quenched at 860 °C (369.2 °C). The M_s_ temperature should be higher in a larger austenite. However, the results of in situ observations of martensitic transformations were extraordinary. The possible reason for this is that the martensitic transformations observed by the in situ method were local to the sample surface, with a limited view field. An unobserved view field may have shown the martensitic transformations at a relatively higher temperature. In addition, it is difficult to unify the different starting temperatures of martensitic transformations due to the uneven composition caused by the evaporation of alloying elements. It was accidentally observed that lath martensite grew through grain boundaries in Figure 12c. These newly formed grain boundaries were relatively straight. In addition, some lath martensite nucleated and grew from the twins. The reason why the twins acted as martensitic nuclei was that martensitic transformations require structural and energy fluctuations. As a kind of crystal defect, twins provide a large defect energy which meets the structural and energy fluctuation requirements. Increasingly more FM and SM gradually appeared with the decrease in temperature, in which the SM appeared at a certain angle to FM. Most martensite transformations finished within 2.25 s, and the rate of martensite transformations slowed down. However, the distortion caused by martensite transformations inhibited the martensite transformations in surrounding austenite (Figure 12j). Martensite growth was a nondiffusion interfacial cooperative pushing process, and the martensite specific volume was larger than that of austenite. Therefore, elastic deformation was caused, accompanied by volume expansion, during the martensite phase transition. Additionally, then a large distortion energy formed, which hindered further martensite transformations.

Martensitic transformations in the same sample did not appear at first in large-sized grains but appeared in the PAGs in a seemingly chaotic manner. This may be explained by the differences in the size, composition, and defect density in PAGs, which led to the selectivity of martensite nuclei. In addition, the grains were coarser due to a higher quenching temperature; thus, the driving force of martensitic transformations was greater. Additionally, the migration rate of phase interfaces increased accordingly, so the martensitic transformations were faster.

**Figure 13:**
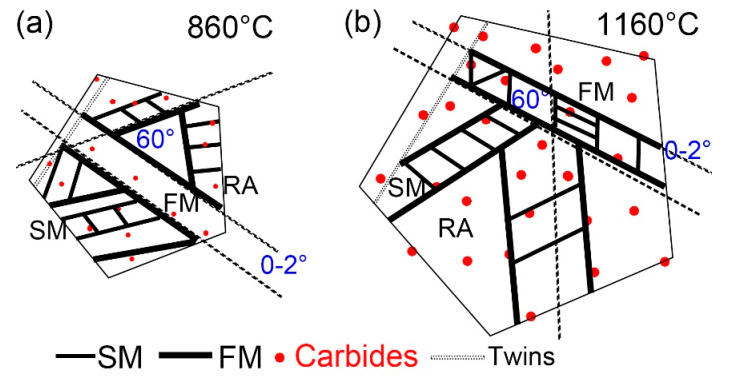
Schematic diagram of martensitic transformations of supercooled austenite at (**a**) 860 °C and (**b**) 1160 °C.

Figure 13 shows the martensitic transformation of supercooled austenite at different quenching temperatures, which is a summary based on in situ observations. Martensite nuclei did not occur simultaneously during the quenching process of supercooled austenite, but selectively proceeded and increased in batches in some areas. This nucleation pattern divided untransformed austenite into multiple regions. In different regions, the size of firstly formed martensite (fresh martensite) was large and the size of subsequent martensite (secondary martensite) was small. This is because the shape of martensite depends on the stress field between the nucleated lath martensite and other martensitic nuclei. The parent austenite presented obvious different grain sizes at different quenching temperatures. In addition, the size and volume fraction of the coarse (Fe, Cr, Mn)_3_C particles in the matrix increased with the quenching temperature.

Martensitic nucleation and growth in different parent austenite grains did not affect each other in the early stage of martensitic transformations, during which less martensite formed. The martensitic transformations gradually increased as the temperature decreased, and the martensitic laths restricted each other. In general, there were three types of martensitic nucleation. Firstly, martensite nucleated along the PAG boundaries and grew in between the grains until stopping when it collided with other lath martensite or grain boundaries. In addition, martensite nucleated at annealing twins, which had lattice defects and provided better structural and energy fluctuations. Moreover, martensite nucleated at the preformed lath martensite and grew in the austenitic grains at about 60° or 120° to form new lath martensite. The lath packet exhibited two types: parallel laths (0~2°) based on the preformed laths and martensitic laths at 60° or 120° in the other direction stimulated by the preformed laths, finally forming triangle, parallelogram, or hexagon morphologies. The formation of SM laths also strongly inhibited the martensitic transformations of the surrounding untransformed austenite and promoted the formation of residual austenite.

## 4. Conclusions

1. The austenite grains in NM500 steel at a quenching temperature of 860 °C (37.41 μm) were smaller than those at a quenching temperature of 1160 °C (119.46 μm). Austenite grains coarsened at ~3 min and ~30 min, respectively, at quenching temperatures of 1160 and 860 °C. In addition, a large amount fine dispersed (Fe, Cr, Mn)_3_C particles redissolved and broke apart at 1160 °C, resulting in many large, visible carbonitrides.
2. The nucleation of martensite did not proceed simultaneously during the quenching process. Selective prenucleation dominated, which divided untransformed austenite into several regions and resulted in a larger size fresh martensite compared to secondary martensite.
3. Martensite can not only nucleate at parent austenite grain boundaries, but it can also nucleate in the preformed lath martensite and twins. The larger the parent austenite grain size, the smaller the constraints of martensite growth, resulting in longer fresh martensite and secondary martensite. In addition, the martensite transformation (2.25 s) was shorter at a higher quenching temperature of 1160 °C than that (13 s) at 860 °C. In addition, martensitic lath could traverse the unstable parent austenitic grain boundaries.
4. The martensitic lath was present in parallel laths (0~2°) based on preformed laths or distributed in triangles, parallelograms, or hexagons with an angle of 60° or 120°.

## Data Availability

The raw/processed data required to reproduce these findings cannot be shared at this time as the data also form part of an ongoing study.

## Glossary

**Words/Equations**	**Abbreviation/Parameter Meaning**
Confocal laser scanning high-temperature microscope	CSLM
Parent austenite grains	PAGs
Fresh martensite	FM
Secondary martensite	SM
$N=f_{N}exp\;(-Q_{N}/K∆T)$ & $G=f_{G}exp\;(-Q_{G}/K∆T)$	N: the nucleation rate; G: the growth rate; $Q_{N}$$\;\mathrm{and}\;Q_{G}$: the nucleation and growth activation energies; $f_{N}$$\;\mathrm{and}\;f_{G}$: the impact factors between structure and nucleation with growth; ∆T: superheat.
$\sum_{i=1}^{i=n}\frac{∆t}{A_{i}}=1$ & $\int_{t=0}^{t=t_{n}}\frac{dt}{A(T)}=1$	∆*t* and *dt*: the transformation time at temperature *T*; *A_i_* and *A*(*T*): the corresponding incubation periods.
$\int_{T_{1}}^{T_{s}}\frac{dt}{A\left(T\right)}=\int_{T_{1}}^{T_{s}}\frac{1}{A\left(T\right)}\cdot \frac{1}{\frac{dT}{dt}}\cdot dT=\int_{T_{1}}^{T_{s}}\frac{1}{A\left(T\right)}\cdot \frac{1}{v}\cdot dT=1$ & $c=\frac{df}{dt},v=\frac{dT}{dt}$ & $\int_{t=0}^{t=t_{n}}\frac{df}{dt}dt=\int_{T_{s}}^{T_{f}}\frac{df}{dt}\cdot \frac{dT}{v}=\int_{T_{s}}^{T_{f}}\frac{C}{v}\cdot dT=1$	*C*: transformation rate; *T_s_* and *T_f_*: the temperatures at the beginning and ending of the transformation; *v*: heating rate; *T*_1_: equilibrium temperature; *t_n_*: the time to the transformation ending temperature *T_f_.*
